# Expansion of the Bactericidal/Permeability Increasing-like (BPI-like) protein locus in cattle

**DOI:** 10.1186/1471-2164-8-75

**Published:** 2007-03-15

**Authors:** Thomas T Wheeler, Kylie A Hood, Nauman J Maqbool, John C McEwan, Colin D Bingle, Shaying Zhao

**Affiliations:** 1 Dairy Science and Technology Section, AgResearch Ruakura, Private Bag 3123, Hamilton, New Zealand; 2 Wakefield Gastroenterology Centre, Wakefield Hospital, Private Bag 7909 Wellington, New Zealand; 3 Bioinformatics, Mathematics & Statistics Section, Invermay, Private Bag 50034, Mosgiel, New Zealand; 4 Animal Genomics Section, AgResearch, Invermay, Private Bag 50034, Mosgiel, New Zealand; 5 Academic Unit of Respiratory Medicine, Division of Genomic Medicine, The University of Sheffield Medical School, M117, Royal Hallamshire Hospital Sheffield S10 2JF, UK; 6 Department of Biochemistry and Molecular Biology, Institute of Bioinformatics, University of Georgia, Athens, Georgia, USA

## Abstract

**Background:**

Cattle and other ruminants have evolved the ability to derive most of their metabolic energy requirement from otherwise indigestible plant matter through a symbiotic relationship with plant fibre degrading microbes within a specialised fermentation chamber, the rumen. The genetic changes underlying the evolution of the ruminant lifestyle are poorly understood. The BPI-like locus encodes several putative innate immune proteins, expressed predominantly in the oral cavity and airways, which are structurally related to Bactericidal/Permeability Increasing protein (BPI). We have previously reported the expression of variant BPI-like proteins in cattle (Biochim Biophys Acta 2002, 1579, 92–100). Characterisation of the BPI-like locus in cattle would lead to a better understanding of the role of the BPI-like proteins in cattle physiology

**Results:**

We have sequenced and characterised a 722 kbp segment of BTA13 containing the bovine BPI-like protein locus. Nine of the 13 contiguous BPI-like genes in the locus in cattle are orthologous to genes in the human and mouse locus, and are thought to play a role in host defence. Phylogenetic analysis indicates the remaining four genes, which we have named BSP30A, BSP30B, BSP30C and BSP30D, appear to have arisen in cattle through a series of duplications. The transcripts of the four BSP30 genes are most abundant in tissues associated with the oral cavity and airways. BSP30C transcripts are also found in the abomasum. This, as well as the ratios of non-synonymous to synonymous differences between pairs of the BSP30 genes, is consistent with at least BSP30C having acquired a distinct function from the other BSP30 proteins and from its paralog in human and mouse, parotid secretory protein (PSP).

**Conclusion:**

The BPI-like locus in mammals appears to have evolved rapidly through multiple gene duplication events, and is thus a hot spot for genome evolution. It is possible that BSP30 gene duplication is a characteristic feature of ruminants and that the BSP30 proteins contribute to an aspect of ruminant-specific physiology.

## Background

Ruminants have acquired a number of physiological and anatomical specialisations in order to adapt to a lifestyle in which pasture is the predominant source of metabolic energy. Most notably ruminants have a fore-stomach, the rumen, in which pasture polysaccharides are broken down by microbial β-glycosidases in a neutral pH anaerobic environment. In addition, ruminants have other adaptations, including a markedly different saliva composition compared with monogastric mammals [1,2]. It is assumed that these physiological adaptations must be accompanied by genetic changes, however, there have been few reports of changes in the genomes of ruminants, which facilitate a specialised ruminant physiological function. Virtually the only such report is of the expansion of the lysozyme locus in cattle [3]. The recent availability of a draft cattle genome sequence, the first for a ruminant, provides an opportunity to discover additional genetic characteristics that facilitate the ruminant lifestyle.

The Bactericidal/Permeability Increasing protein (BPI) plays an important role in host-defence in mammals. BPI is found in the secretory granules of neutrophils and is secreted in response to activation of Toll-like receptor (TLR)-mediated signalling, whereupon it acts as an innate immune effector protein by permeabilising the plasma membrane of Gram negative bacteria as well as attenuating the TLR response [4,5]. Three well-characterised proteins have some sequence conservation with BPI. Lipopolysaccharide binding protein (LBP) is secreted from the liver into the circulation where it appears to act as a sensor for the presence of bacteria [6]. LBP acts as an opsin, binding lipopolysaccharide (LPS) derived from the outer membrane of Gram negative bacteria, and thence stimulating a TLR-mediated innate immune response [7]. Phospholipid transfer protein (PLTP) and cholesteryl ester transfer protein (CETP) function as lipid transport proteins in the blood (reviewed in [8,9]). Recent reports have shown the existence of at least 10 additional genes in humans and mice, which are related to BPI through sequence similarity, exon segmentation and predicted secondary structure [10,11]. All but two of these are found as a gene cluster at a single locus on human chromosome 20 or the syntenic region of mouse chromosome 2. The similarity of the products of these genes to BPI and LBP, their expression in oral cavity and airways tissues [12-16] and evidence for the antimicrobial activity of at least one of them [17] suggests that they play a role in host defence.

We have previously characterised two closely related members of the expanded BPI-like protein family in cattle. These proteins, BSP30A and BSP30B, are expressed in saliva and are both most closely related to human and mouse PSP [18-20]. We have now characterised the entire BPI-like protein locus in cattle in order to understand the relationship of the BSP30A and BSP30B genes to one another and to PSP, and to understand the evolutionary events that have occurred in the locus in cattle, particularly gene duplication. Here we report that the bovine locus contains 13 BPI-like genes, comprising nine homologues of BPI-like genes from mouse and human as well as four paralogues of PSP, two of which have not been previously described. These appear to have arisen from a series of gene duplication events. Their distinct patterns of transcript abundance, and their presence in at least some other ruminant species is consistent with the multiple BSP30 genes having a specific role in ruminant physiology.

## Results

### Characterisation of the bovine BPI-like locus

Six bovine BACs spanning the BPI-like locus were identified by alignment of bovine BAC end sequences with the region of the human genome sequence containing the BPI-like locus. These BACs were subjected to random shotgun high throughput sequencing. These sequences, as well as sequence contigs from the bovine assembly (BosTau 2.0, current at the time study was undertaken) were used to create a single 721,869 bp contig as described in the Methods [GenBank: DQ667137].

**Figure 1:**
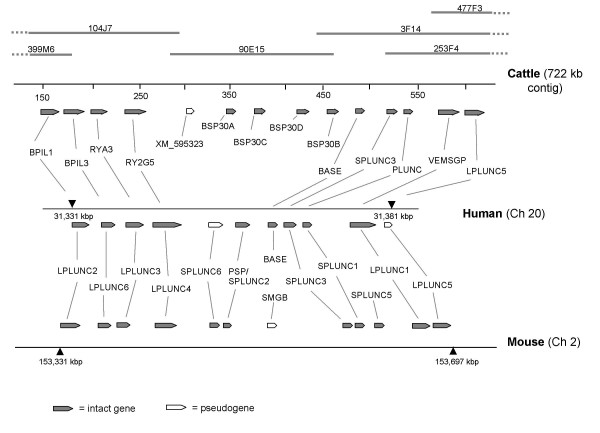
**The BPI-like protein locus in cattle, human and mouse**. The position on the chromosome is indicated by nucleotide number (in small font) of the fully assembled human (hg17) and mouse (mm6) chromosomes, obtained from the UCSC Goldenpath genome browser [48]. The cattle coordinates are that of the 759 kbp assembled contig. The shaded and solid bars above the cattle contig indicate the positions of the sequenced BACs. The arrowed end of each gene indicates the direction of the ORF. Intact genes are shaded while pseudogenes are not filled.

To identify the genes within the bovine genomic contig, sequences from human and bovine RefSeq, and TIGR bovine Gene Indices, along with the known bovine BPI-like gene sequences from GenBank and Ensembl bovine gene predictions, were mapped onto the contig using the GMAP and Exonerate programs. In total, 21 putative genes or pseudogenes were identified. Of these, 14 contiguous genes spanning 470 kbp appeared to be related to members of the BPI-like family (listed in Table 1). The gene order obtained resembled that of the previously characterised human and mouse BPI-like loci [14,21] much more closely than that of the BosTau v2.0 assembly (Figure 1), due to assembly errors in the ver 2.0 of the bovine assembly. Among the 14 genes, only BSP30A, BSP30B, PLUNC and VEMSGP have been previously described and characterised in cattle [19,20]. For two of the genes (BSP30C and SPLUNC3), cDNA sequence was obtained by sequencing of individual cDNA clones and extension using 5' and 3' RACE. After re-sequencing, a correction was made to the previously determined BSP30A sequence (GenBank accession number U79413). For one of the genes (BPIL1) the Ensembl bovine gene prediction was found to be the putative cDNA sequence that better matched homologues in other species. For three of the genes (BPIL3, RYA3 and RY2G5) a better match to other species was obtained by combining the Ensembl and RefSeq predicted sequences. For two other genes (BASE and LPLUNC5) the final cDNA sequences were produced by the Genscan gene prediction program. These sequences (see Additional file 1 for details) were aligned with the genomic contig using the Spidey program [22] to determine the position, number and size of the exons (Table 1).

Alignment of the bovine BPI-like predicted amino acid sequences revealed significant similarity with the known BPI-like genes from human and mouse. Nine of the 14 bovine BPI-like genes were orthologous to genes in these species as they formed reciprocal pairs of top BLAST hits between species (see Table 1 for % amino acid identities). The remaining five are most similar to PSP from mouse and human, for which there is no unambiguous ortholog in bovine (see Table 2 for % amino acid identities). Two of these have been previously described as BSP30A and BSP30B [19]. We therefore named the two additional apparently complete genes BSP30C and BSP30D. The fifth, (bovine RefSeq entry XM_595323) is substantially truncated compared with other BPI-like genes. It contains an open reading frame containing 121 amino acids encoding a 13 kDa protein and comprising three exons of 113, 145 and 104 nucleotides in length, and has no TATA box or CpG islands associated with it. Therefore, it is most likely a pseudogene. All 13 intact bovine BPI-like genes conform to the expected structure of BPI-like proteins, having a predicted mass of either approximately 27 kDa or 53 kDa, having either 6–9 or 15–17 exons, and having either one or two BPI domains. These predicted amino acid sequences include a secretion signal sequence of 20 amino acids at the N-terminus. The subset of BPI-like proteins of approximately 27 kDa having one BPI domain have been referred to as SPLUNC1-4 and the approximately 53 kDa proteins with two BPI domains as LPLUNC1-5 [21]. Two conserved cysteines that have been shown to form a disulphide bond between amino acid 135 and 175 of human BPI [23] are present in all the bovine BPI-like proteins, and are separated by 35–46 amino acid. The bovine BASE and LPLUNC5 genes, which appear to be pseudogenes in human, appear to be fully intact genes in cattle. No bovine homologues were found for the mouse BPI-like genes SMGB, SPLUNC5 or SPLUNC6.

### Expression of the BPI-like genes in cattle

Each of the predicted bovine BPI-like cDNA sequences were used as query to search for matches to experimentally produced cDNA sequences contained in an AgResearch database of ESTs [GenBank: DY037420-DY223196] as well as the TIGR bovine Gene Indices. Positive matches were obtained for 7 of the 13 bovine BPI-like genes indicating they are expressed (Table 3). Alignment of the assembled EST contigs with the predicted cDNA sequences were used to refine the cDNA sequences for BPIL1, BSP30C and SPLUNC3 as described in the Methods.

**Figure 2:**
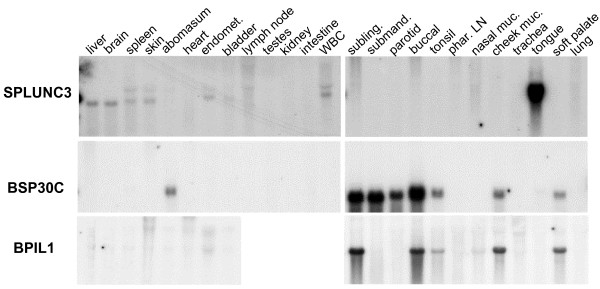
**Tissue-specific expression of BPI-like genes**: Northern blots were loaded with RNA from the following bovine tissues: (1) liver, (2) brain, (3) spleen, (4) skin, (5) abomasum (6) heart, (7) endometrium, (8) bladder, (9) lymph node, (10) testes, (11) kidney, (12) small intestine, (13) white blood cells, (14) sublingual salivary gland, (15) submandibular salivary gland, (16) parotid salivary gland, (17) buccal salivary gland, (18) tonsil, (19) pharyngeal lymph node, (20) nasal mucosa, (21) cheek mucosa, (22) trachea, (23) tongue, (24) soft palate, (25) lung. The blots were probed with full length cDNA encoding SPLUNC3 (top panels), BSP30C (middle panels) and BPIL1 (bottom panels).

We have previously demonstrated that four members of the BPI-like protein family in cattle (BSP30A, BSP30B, PLUNC and VEMSGP) are found in a restricted range of tissues associated with the bovine oral cavity and airways [19,20]. The pattern of transcript abundance of three additional BPI-like protein genes in cattle was determined by Northern blotting using RNA extracted from a range of bovine tissues. The results showed that BSP30C, SPLUNC3, and BPIL1 are each expressed to a relatively high level in a restricted range of bovine tissues (Fig. 2). BSP30C mRNA was found in salivary glands, nasal mucosa, tongue and abomasum, while a high abundance of SPLUNC3 transcripts was found only in tongue. BPIL1 transcripts were found to be expressed only in the sublingual and buccal salivary glands, tonsil, cheek epithelium, and the soft palate.

**Figure 3:**
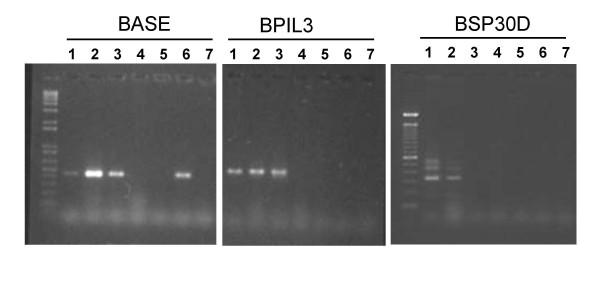
**Abundance of transcripts for BPI-like genes in different tissues**: Reverse transcriptase-PCR analyses were performed using RNA extracted from the following bovine tissues: (1) parotid salivary gland, (2) submandibular salivary gland, (3) buccal salivary gland, (4) tracheal lining, (5) olfactory tract mucosa, (6) nasal mucosa, (7) vomeronasal organ. The PCR was performed using primer pairs specific for bovine BASE and BPIL3, as indicated. The primer sequences are listed in the Methods. The expected product sizes are 596 bp (BPIL3), 454 bp (BASE), and 428 bp (BSP30D).

Evidence for expression of additional BPI-like proteins was obtained using RT-PCR. Specific amplification was obtained using primers derived from bovine BASE, BPIL3 and BSP30D. The nucleotide sequences of these PCR products were obtained [GenBank:DQ777771 , DQ777772 and DQ777773] and were found to align with the predicted cDNA sequences. BASE and BPIL3 cDNAs were amplified from salivary tissues, and in addition BASE cDNA was amplified from the nasal mucosa. Three distinct bands, each containing BSP30D cDNA, were amplified from parotid and submandibular salivary tissue (Fig. 3). The multiple bands are most likely due to variable splicing of BSP30D mRNA. In total, evidence was obtained for the expression of 11 of the 13 BPI-like genes in cattle (Table 3).

### Evolution of the BPI-like locus

**Figure 4:**
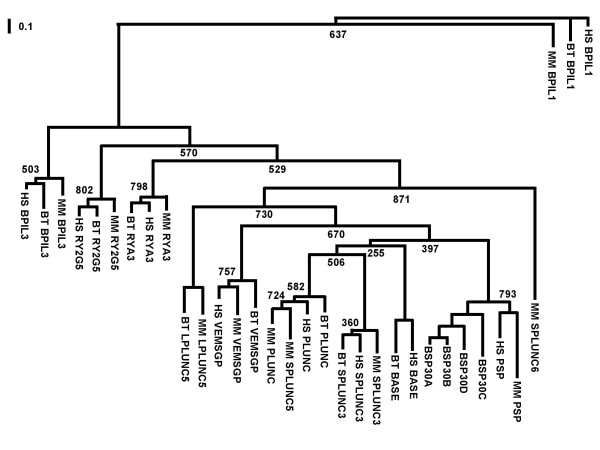
**Phylogenetic analysis of the BPI-like genes**: A consensus Maximum-Likelihood tree was generated using the PHYML program after aligning the nucleotide sequences from the N-terminal domain of the two-domain and the full length sequences of the single-domain BPI-like proteins. The values associated with the branch nodes indicate the level of support derived from bootstrap analysis of 1000 replicates. The bar indicates 10 nucleotide substitutions per 100 sequence positions. The sequences used were from human (HS), mouse (MM) or cattle (BT) (see Table 1, Additional file 1 and Additional file 2).

Cattle is the third species, the others being human and mouse [14,21], for which the complete BPI-like locus has been described. As a first step in understanding the evolutionary relationships among the members of the BPI-like family, the sequences from the intact genes within the BPI-like locus in cattle, human and mouse were aligned and a phylogenetic tree was constructed (Fig. 4). This tree confirms the status of nine of the bovine genes as orthologs of genes in other species, and indicates that the four BSP30 genes are clearly most closely related to PSP. In addition, the four BSP30 genes are part of a sub-group comprising all the single-domain proteins (the short PLUNCs). Of all the two-domain proteins, the VEMSGP gene is most closely related to the single-domain proteins.

**Figure 5:**
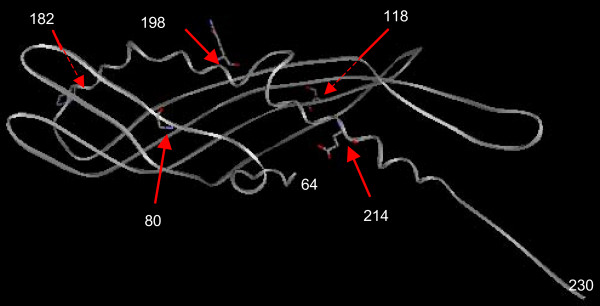
**Predicted protein fold for BSP30A**: A secondary structure view is shown of the fold predicted for BSP30A by the PHYRE protein threading program, produced as described in the Methods. The amino acids identified by Codeml as being under evolutionary pressure for divergence between the BSP30 and PSP genes, listed in Table 6, are indicated. These amino acids are viewed as a wire representation.

In order to determine the extent of evolutionary pressure on the BPI-like proteins, the ratio (ω = *d*_N_/*d*_S_) of non-synonymous (*d*_N_) to synonymous (*d*_S_) substitutions was calculated between orthologous pairs of intact BPI-like genes as well as between the BSP30 and PSP genes. The ratios were less than 1 for all but two of the 22 pairs of BPI-like orthologues (Table 4). This indicates that there has been evolutionary pressure for amino acid sequence conservation in these genes since the divergence of human, mouse and cattle. A similar analysis of the four BSP30 genes together with human and mouse PSP genes resulted in ratios not significantly different from 1 between pairings of the four BSP30 genes and either human or mouse PSP. This indicates relaxation of pressure for amino acid sequence conservation (Table 5). To determine if this was due to positive selection for divergence or solely relaxed selection for conservation i.e. if the ω values were different between the bovine BSP30 and human and mouse PSP genes, two codeml branch models were fitted, one where the bovine BSP30 genes were allowed to have one ω ratio and the PSP genes to have another ratio (two-ratio model) and second where all BSP and PSP genes had the same ratios (one ratio). The likelihood of the model with different ratios (LnL = -4057.73) was found to be significantly better (p-val < 0.05) than the one-ratio model (LnL = -4060.12), showing the evidence of positive selection. In addition, ratios between BSP30C and the other BSP30 proteins were significantly greater than 1, indicating that there is positive selection pressure on BSP30C for divergence of its amino acid sequence from the other BSP30 proteins. To identify the amino acid sites under positive selection, four different models M1a (NearlyNeutral), M2a (PositiveSelection), M7 (β) and M8 (β and ω > 1) were tested. The results are shown in Table 6. Both the M2a and M8 models for selection were significantly better than M1a and M7 respectively, with evidence for a proportion of sites (ca. 20% for both models) under positive selection. The two models also identified six identical amino acid sites with Bayesian probabilities of > 0.95 to have the ω > 1 (listed in Table 6). Protein threading analysis using the PHYRE program [24] resulted in a very good fit (expect value <E^-9^) for BSP30A onto the structural model of the N-terminal domain of BPI, despite its less than 40% amino acid sequence identity. This suggests that among the bovine BPI-like proteins, at least BSP30A has the same protein fold as BPI, including a similar hydrophobic pocket. The six sites under positive selection were mapped onto the structure of BSP30A (Fig. 5). Five of the six sites are in positions that are likely to contribute to the shape of the hydrophobic pocket. It is possible that the hydrophobic pocket functions to bind a substrate. Therefore, changes in these sites may influence the binding specificity of the protein.

**Figure 6:**
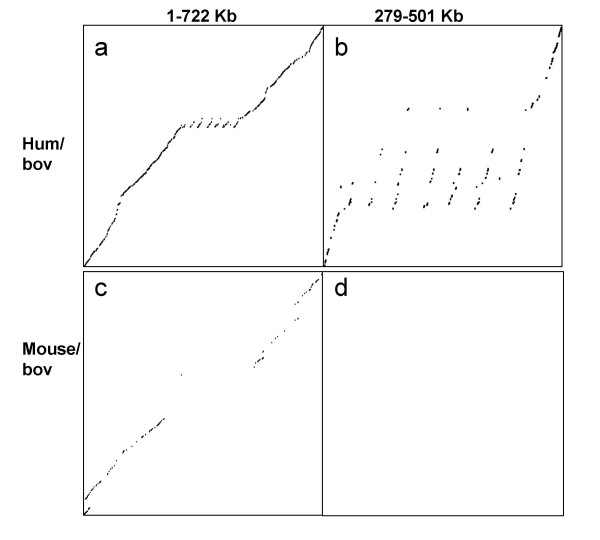
**Dot-plot pairwise alignment of the BPI-like locus between species**: The genomic nucleotide sequence in mouse and human were aligned using the OWEN program as described in the Methods. The top panels show the cattle – human alignment while the bottom panels show the cattle – mouse alignment. The left hand panels show the alignment across the full locus, while the right hand panels show the area of the locus containing the cattle duplications in more detail.

**Figure 7:**
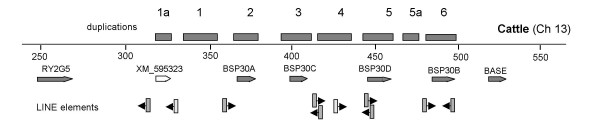
**Mapping of the duplications onto the cattle BPI-like locus**: The position of the genomic duplications is shown as shaded boxes above the line. The position of the intact genes is indicated by shaded arrows while the pseudogene is indicated by an unshaded arrow. The stippled bars with arrows indicate the position and direction of the L1Bt LINE element while the shaded vertical bars with arrows indicate the position and direction of the RTEBt1 LINE element.

The contiguous genomic sequence of the bovine BPI-like locus was aligned with the orthologous region from human using a dot-plot algorithm. For most of its length, the sequences from both species align approximately1:1. However, a 165 kbp region of the bovine contig (between 300 kbp and 465 kbp) contained a series of eight repeats, ranging in size from 4 to 22 kbp (Fig. 6). Four of the eight repeats contained the BSP30A, BSP30B, BSP30C and BSP30D genes within them. One of the remaining four repeats contained the pseudogene identified during initial characterisation of the locus (XM_595323) (Fig. 7). A gene prediction analysis of the other three repeats resulted only in gene fragments. A similar analysis comparing the bovine sequence with that of mouse revealed no such repeats, but instead showed a large segment of the locus that did not align (Fig. 6). A dot-plot alignment of the bovine BPI-like locus genomic sequence against the equivalent region of the dog genome revealed a similar series of eight repeats (results not shown). The interspecies alignments showed that the repeated section in bovine is not present in the mouse genome, which is significantly diverged in this region.

A segment of the genomic contig from the RY2G5 to the BASE gene was used as query in a BLAST search of a bovine repetitive sequence database [25] to search for the presence of repeat elements within it. The search returned two Long Interspersed Nuclear Elements (LINEs), L1Bt and RTEBt1. In total, significant alignments of greater than 800 bp were obtained at ten locations along the genomic segment. All but one of these were positioned within or very close to the gaps between the genomic duplications (Fig. 7). It is possible that these LINE elements could have contributed to the genome instability in this region of the locus, resulting in the multiple gene duplications, in a manner analogous to what has previously been suggested for Alu sequences in the human genome [26].

## Discussion

This report provides the first characterisation of the locus encoding the BPI-like proteins in a ruminant species. The analyses have shed light on how the locus has evolved as well as raised possibilities regarding the function of the BPI-like proteins in ruminants. The structural similarity and clustering of the individual genes in the BPI-like locus in the bovine, mouse, and human genomes suggests evolution from a single ancestral gene, with gene duplication followed by divergent evolution giving rise to the differences between the family members. This appears to be particularly prevalent in the bovine BPI-like protein locus compared with the other species. Gene duplication has been previously noted in ruminants. Duplication of a single ancestral secretory RNAse gene appears to have given rise to the separate pancreatic, seminal and brain RNAse genes found in ruminants [27]. Ruminants and other artiodactyls contain a large number of genes encoding the pregnancy-associated glycoproteins, which appear to have arisen by gene duplication [28]. Duplication of the lysozyme gene is a feature in its recruitment as a digestive enzyme in ruminant artiodactyls [3]. The orientation of the genes in the BPI-like cluster is consistent with gene amplification by a series of unequal crossovers [29]. The presence of multiple LINE elements within the expanded BSP30 region of the locus provides one possible mechanism for the initiation step of the expansion.

The family of proteins in the cluster is divided into those with one or two BPI domains (the long and short PLUNCs [21]). The single-domain genes are contiguous within the cluster. It is possible that the ancestral BPI-like gene was a two-domain lipid binding protein. The six two-domain proteins then arose through a series of gene duplications. The first single-domain protein may have been created through duplication of the N-terminal portion of one of the two-domain proteins. The phylogenetic analysis together with their position in the locus suggests that this is most likely to have been the VEMSGP gene giving rise to the PLUNC gene. Under this scenario, subsequent duplication of PLUNC would have given rise to additional single-domain proteins, some of which could have been duplicated in turn, as appears to be the case with PSP giving rise to the four BSP30 genes. The most recent duplication appears to have been that giving rise to BSP30A and B. The data indicates that duplication events have occurred more frequently than the minimal number of times required for the observed gene duplications. Interestingly, it appears that there have been distinct duplications in the mouse lineage giving rise to SPLUNC5 and SPLUNC6.

The duplications giving rise to the four BSP30 genes in cattle may have occurred after divergence of cattle from other mammals. Examination of genome assemblies from other species indicates that human, dog and chimpanzee have only a single PSP ortholog. Furthermore, a search of EST databases revealed only single PSP orthologs in human, dog and pig [GenBank:CB986486, DN405753 and CJ027526, respectively]. Analysis of an in-house sheep EST database revealed four ESTs [GenBank: EE792560, EE792910, EE794201, and EE794362] that align closely with both BSP30A and BSP30B. This indicates that homologs of BSP30A and/or B exist in a second ruminant species, the sheep, consistent with the possibility that the BSP30 gene expansion may have coincided with the radiation of ruminant species. Further analyses are required to confirm this.

The analyses reported here raise some intriguing questions regarding the function of the BPI-like proteins. All of the greater family of BPI-like proteins characterised to date share a common biochemical property in binding complex lipids. These proteins have distinct sites of expression and diverse functions such as lipid transport and innate immunity. This and other reports [14,16,20,30] show that most of the BPI-like proteins are most abundant in tissues associated with the oral cavity and airways. Here we show that at least one family member is also expressed at a lower level in the digestive tract. It is likely the BPI-like proteins function in epithelial mucosa after being secreted.

The question of whether the BSP30 proteins have a similar lipid binding or bactericidal activity to that of BPI awaits experimental verification. In support of this, recombinant human PSP has been reported to inhibit the growth of *P. aeruginosa *[17], and human PLUNC, has been shown to bind LPS [31]. If these activities for the BSP30 proteins are confirmed, a possible biological role for them could be in modulating the microbial ecology in the bovine oral cavity so as to maintain optimal digestive function or prevent pathological infection. The results presented here suggest that the shape of the hydrophobic pocket may be important for determining functional differences among the BSP30 proteins.

## Conclusion

The bovine BPI-like locus of cattle features expansion of the single PSP gene present in human and mouse into four distinct BSP30 genes. The *d*_N_/*d*_S _ratio data are consistent with evolutionary pressure for conservation of protein structure between all the orthologous pairs of BPI-like genes in cattle, human and mouse. However, this pressure is absent between the BSP30 genes, and the data suggests there is pressure for sequence divergence between BSP30C and the other BSP30 proteins. This, as well as its distinct expression profile, is consistent with BSP30C having acquired a distinct function from the other BSP30 proteins. The most likely biological role of the BPI-like proteins, including the BSP30 proteins, is as either detector or effector proteins in innate immune host defence [11]. While their precise biological roles are unknown, one can speculate that the BSP30 proteins may influence the host response to the commensal microbial ecosystem in cattle, including that of the rumen. BSP30A and B comprise approximately 30% of total salivary protein in cattle [18], thus resulting in up to 150 g per day of BSP30 proteins being delivered into the rumen. A focus for future investigations is to determine whether the BSP30 gene duplications and subsequent divergence observed in cattle could have been a key step in the evolution of ruminants by facilitating adaptation to a ruminant lifestyle.

## Methods

### BAC screening, sequencing and assembly

A 1 Mbp region of the human assembly containing the PSP region was downloaded from UCSC (hg16:Chr20:32–33 Mbp lower case masked) and compared against 292,638 reads from the ends of individual random BAC clones derived from two bovine genomic DNA BAC libraries (downloaded NCBI Nov 2003) using BLASTN and the following options -m 8 -e 1e-2 -U T. The output was then processed and ranked for high quality paired end hits in the appropriate orientation and estimated size. Over 30 BAC clones mapped to the human BPI-like locus on chromosome 20. These were screened for the presence of known BPI-like genes by PCR using primer sequences derived from the previously determined bovine cDNA sequences of BPIL1, VEMSGP, PLUNC, BPIL3 and RYA3. The primer pair for BSP30B was derived from sequencing of a segment of the BSP30B promoter contained within a bovine genomic DNA clone in a cosmid vector. The following primer pairs were used. BSP30B: CACATCCTCACCACACACCTGGA and CAGACTGTCTGTGTCCAGTTCTGC; BPIL1: AGTTTCCCGAGCCCATGCCT and GGACTGGAAAGCCGAGTTGGAG; VEMSGP; GCCAGGTTGTTCAACTCAGAA and GTGAGTTTTCCCGAATGG; PLUNC: CTCTCAGCAATGGCCTGCTCT and GGAGAGGGGTGAGTGAAGTCACTT; BPIL3: TGCTGGCTTCTCCAGGCTGT and AAGCAGCCCCCACCACTCAA; RYA3: ACACTGCCTCTCATCTCCAACCA and AGGTTTAGCCAAGTAGAGGCCATT. The PCR products were gel purified, cloned into pGEM-Teasy vector (Promega) and sequenced to confirm the specificity of the PCR screen.

Six BAC clones that were confirmed by PCR to contain parts of the BPI-like locus (CH240_399M6, CH240_104J7, CH240_90E15, CH240_3F14, CH240_477F3, and CH240_253F4) were selected for high throughput shotgun sequencing. This was performed by the TIGR library construction, random sequencing, and closure teams as follows. BAC DNA was isolated, nebulized, the ends polished, and adaptors added. The DNA were size-selected (2–3 kbp and 8–10 kbp), ligated to a modified pBR322 vector, and transformed into *E. coli *[32]. The libraries were checked for insert size, bovine origin, randomness, and overlap between clones prior to high throughput sequencing. Templates were then prepared from the shotgun clones using an automated production pipeline. Sequencing reactions were carried out on plasmid templates with MJ Research thermocyclers using Applied Biosystems PRISM Big Dye™ Terminator Cycle Sequencing Ready Reaction Kits. Reactions were set-up by Beckman Multimek automated pipetting robotic workstations combining templates and reaction mixes. Thirty to forty consecutive cycles of linear amplification steps were performed. The reactions were then cleaned up by ethanol precipitation and analysed on Applied Biosystems 3730xl DNA Analyzers. Base calling was performed with phred and Paracel TraceTuner that had been trained with TIGR trace data. Sequence trimming was conducted using LUCY – a program developed at TIGR [33] with a trimming standard of an overall base call error rate of <1%, free of vector- and *E. coli *sequences, and a trimmed sequence read length of > 100 bp.

A total of 11,445 vector screened and clipped shotgun sequences, with Phred quality scores, were assembled using Phrap resulting in eight contigs ranging in size from 2 kbp to 158 kbp. The contigs were then mapped on to the version 2.0 of the bovine assembly (BosTau2.0, The Bovine Genome Sequencing Project Consortium) and the length of the between-contig gaps were estimated (which ranged between 79 to 519 bp, while one gap could not be estimated with the current depth of the genomic assembly). The final sequence was submitted to HTG part of the GenBank (accession number DQ667137). The ver 2.0 of the bovine genomic assembly was found to have order and orientation anomalies, which is quite common in the draft assemblies. However, the underlying contigs were found to be assembled correctly. Thus the between contig gaps were filled in from the bovine genomic assembly.

### Gene mapping

Known cDNAs and protein sequences of the BPI-like genes from human, mouse and cattle from the GenBank, Ensembl bovine gene and protein predictions of the BPI-like genes [34] the human and bovine RefSeq nucleotide and protein sequences (release 13) and the TIGR bovine Gene Indices (release 11.0) [35], were mapped on to the 722 kbp bovine genomic contig. The nucleotide sequences were mapped using the GMAP program [36], while the protein sequences were mapped using protein2genome model of Exonerate [37]. The bovine genomic contig was also used as query to predict additional genes within the BPI-like locus using the Genescan gene prediction program [38].

The 722 kbp genomic contig was used as a backbone on an in-house installation of the Generic genome Browser, [39]. The contigs resulting from the BAC sequencing, gene mappings as well as the gene predictions were then put as tracks on the GBrowse to facilitate identification of members of this family.

### Sequencing of SPLUNC3, BSP30A and BSP30C cDNA

The predicted cDNA sequences for SPLUNC3, BSP30A and BSP30C were used to query an in-house database of over 200,000 bovine ESTs. These searches resulted contigs that matched very well to each of the genes, but whose sequence differed slightly from the predicted sequence. For SPLUNC3 and BSP30C, the contig sequence was extended using 5' and 3' RACE [GenBank: DQ677839 and DQ835286]. For BSP30A, additional clones were obtained by RT-PCR and the region of sequence divergence with the previous GenBank entry [U79413] was sequenced. These additional clones had identical sequence to the EST contig, thus confirming a reading error in U79413. The updated sequence was submitted to GenBank [U79413]

### Sequence analysis

The protein sequences of the bovine, human and mouse BPI-like genes were aligned using the SATCHMO (Simultaneous Alignment and Tree Construction using Hidden Markov mOdels) [34] with a window size of 13. The C-terminal domains of the two-domain proteins (i.e. long PLUNCs) were removed before aligning them to ensure appropriate alignment between the one- and two-domain proteins. The SATCHMO alignment of the protein sequences was converted into a nucleotide alignment using TRANALIGN program of EMBOSS [35]. An unrooted tree was constructed using the maximum likelihood method in PHYML v2.4.4 [40] with bootstrap support, at the nodes, computed for 1000 replications of the data. A general time-reversible model of DNA substitution (GTR) was used in the maximum likelihood and the initial tree used was BIONJ [41].

The rate of nonsynonymous (*d*_N_) and synonymous (*d*_S_) substitutions was calculated following the method of Yang and Nielsen [42] as implemented in the program *yn*00 which is a part of PAML package (Phylogenetic Analysis by Maximum Likelihood ver 3.15) [43]. Codeml, also part of the PAML package, was used to detect the positive selection for amino acid sequence divergence in the bovine BSP30 versus human and mouse PSP genes. The reduced alignment of the bovine BSP30 and human and mouse PSP genes were fitted with two maximum likelihood models, namely one ratio and two-ratio models. The one-ratio model assumed ω to be equal for all the branches in the reduced tree, while the two-ratio model allowed one ω value for the bovine branches and a possibly different value of ω for the Human and Mouse PSP branches. The test of differences between different models was carried out using a chi squared test. The evidence of positive selection was obtained where the two-ratio model was found to be significantly better than the one-ratio model, with ω estimated to be higher in the bovine lineage.

A sites model was also fitted to the reduced alignment of bovine BSP30 and human and mouse PSP genes to identify the amino acid residues under selection for divergence. The models implemented in codeml, namely, M1a, M2a, M7 and M8 were fitted for this purpose. The likelihood ratios from M1a and M2a and M7 and M8 were compared for the evidence of selection for divergence among sites. Bayesian probabilities [44] were calculated for each amino acid in the alignment, after removing ambiguity characters and those with Prob(ω >1) > 0.95 are reported.

For the genomic alignments, sequences were retrieved from hg17:chr20: 30,700,000–31,700,000, mm7:chr2: 153,400,000–154,400,000 and canFam2:chr24:24,900,000–25,900,000 and aligned with the assembled bovine region using OWEN [45]. All sequences were assembled with the same parameters which consisted of an initial round using default parameters and then a second round with a lower threshold (1E-4) followed by manual removal of the off diagonal elements.

### mRNA analysis

A range of bovine tissues were obtained from a Friesian-Holstein dairy cow at slaughter, snap frozen in liquid nitrogen and ground to a frozen powder in liquid nitrogen. RNA was isolated from the tissues using Trizol (Invitrogen) following the manufacturer's instructions. RNA was resolved on a formaldehyde-agarose gel, transferred to membrane and probed with ^32^P-labelled cDNA as previously described [19]. The probes used were full length bovine BSP30C, SPLUNC3 and BPIL1 cDNA. The blots were washed at moderately high stringency (65°C in phosphate buffer [46]) and the signal was visualised by exposure to X-ray film.

Reverse transcriptase polymerase chain reaction (RT-PCR) was performed on RNA isolated from a similar range of bovine tissues to that described above. RT reactions were performed using 1 μg of RNA and MMLV reverse transcriptase in a 20 μl reaction following an established protocol [47]. A 1 μl aliquot was subjected to PCR using the following primer sets: actin; CGCACCACTGGCATTGTCAT and TTCTCCTTGATGTCACGCAC, BPIL3; CCAGGGATGAAGCCTATCAA and TGTGAGGAGCCTTCAGCATA, BASE; GAAGGTCTCCAGCCTCTTCA and CTCAGGAATGAGCCTGCAAT, BSP30D; TGAGGCGGACCCAGAGAAGA and AATGCGTTACCAGGGACAATAC. The actin, BASE and BPIL3 reactions employed an annealing temperature of 55°C and proceeded for 30 cycles. The annealing temperature of the BSP30D reaction was 60°C. The amplified DNAs were resolved on agarose gels and visualised by staining with ethidium bromide.

### Structure prediction

The BSP30A amino acid sequence was submitted to the on-line protein threading program, PHYRE [24]. The search returned a top structure prediction with an expect value of 2.2e-09, with a precision value of 100%. The thread was based on the crystal structure of BPI. The structure was viewed using the 3D molecule viewer module of the Vector NTI software package.

## Authors' contributions

TW proposed the research goal, supervised the analyses, performed the RT-PCR studies and wrote the manuscript. KH carried out the BAC screening, cDNA sequencing, Northern analyses and provided a critical review of the manuscript. NM performed the contig assembly, gene mapping, phylogenetic and Ka:Ks analyses, and wrote parts of the manuscript. JM contributed to the design of the study, organised and supervised the BAC screening, sequencing and contig assembly, and wrote parts of the manuscript. CB contributed to the gene mapping, contributed to the interpretation of results and provided a critical review of the manuscript. SZ organised the BAC sequencing. All authors read and approved the final manuscript.

## Supplementary Material

Additional file 1 Final nucleotide and amino acid sequences for the BPI-Like genes, not already in the public sequence databases. The N-terminal domain of the two-domain (all but BSP30C, BSP30D and BASE) and the full length sequences of the single-domain sequences (BSP30C, BSP30D and BASE) were used in the Phylogenetic analyses Click here for file

Additional file 2 List of public domain nucleotide sequences (The N-terminal domain of the two-domain and the full length sequences of the single-domain sequences were used for the phylogenetic analysis). Click here for file

## Figures and Tables

**Table 1:** Genes comprising the bovine BPI-like locus

Gene	Alt. Names^1^	Access. Number	cDNA length(bp)	length aa	pred. MW	# exons	start of first exon	end of last exon	% aa identity^2^
BPIL1	LPLUNC2, Z	ENSBTAG00000019200^3,7^	1380	459	49	15	145,091	163,442	73 (h), 63 (m)
BPIL3	LPLUNC6, Y	XM_596762^5,7^	1332	443	49	17	168,578	187,389	79 (h), 68 (m)
RYA3	LPLUNC3	XM_600293^5,7^	1434	477	50	15	196,342	213,257	80 (h), 82 (m)
RY2G5	LPLUNC4	XM_593919^5,7^	1722	574	60	15	222,648	256,128	84 (h), 85 (m)
XM_595323		XM_595323	362	121	13	3	301,883	304,761	
BSP30A		NM_174803	976	239	26	9	343,495	354,041	
BSP30C		TC277047^4,7^	1180	251	28	8	374,201	383,493	
BSP30D		Genescan^6,7^	694	231	25	6	421,589	432,785	
BSP30B		NM_174802	980	240	27	9	453,354	463,730	
BASE	Latherin	Genescan^6,7^	687	229	26	7	484,319	495,374	42 (h)
SPLUNC3	X	DQ677839	1029	239	27	7	516,034	527,454	67 (h), 63 (m)
PLUNC	SPLUNC1	NM_174426	1049	255	26	9	536,029	543,093	73 (h), 65 (m)
VEMSGP	LPLUNC1	NM_174697	1624	473	52	15	573,091	594,293	56 (h), 49 (m)
LPLUNC5		Genescan^6,7^	1551	517	57	15	600,122	618,771	50 (h), 59 (m)

(1) [10, 19]

(2) Amino acid sequence identity obtained using ClustalW against either the human (h) or mouse (m) ortholog.

(3) Ensembl database [34]

(4) TIGR database [35]

(5) Bovine RefSeq predicted coding sequence trimmed to align with human and mouse homologs

(6) Predicted cDNA sequence generated from genomic contig using Genescan)

(7) See supplementary material sTable 1 for final nucleotide and amino acid sequences used in the analyses

**Table 2:** alignment of BSP30 and PSP amino acid sequences (% aa identity)

BSP30A	BSP30B	BSP30C	BSP30D	hum PSP	mouse PSP	
-	83	39	51	39	27	BSP30A
	-	37	50	38	25	BSP30B
		-	35	32	23	BSP30C
			-	37	30	BSP30D
				-	32	hum PSP

**Table 3:** Expression of bovine BPI-like genes (Y indicates expression observed)

Gene	GenBank accession	TIGR Gene Indices	Northern	RT-PCR
BPIL1	DQ835287	-	Y	
BPIL3	DQ777772	-		Y
RYA3	-	-		
RY2G5	-	BE754700		
BSP30A	NM_174803	TC283560	Y^1^	
BSP30C	DQ835286	TC277047	Y	
BSP30D	DQ777773	-		Y
BSP30B	U79414	TC299635	Y^1^	
BASE	DQ777771	-		Y
SPLUNC3	DQ677839	-	Y	
PLUNC	NM_174426	TC279567	Y^2^	
VEMSGP	NM_174697	TC280967	Y^2^	
LPLUNC5	-	-		

(1) [19]

(2) [20]

**Table 4:** dN/dS ratios (± SE) for the BPI-like protein genes

	Bov/hum	Bov/mouse	hum/mouse
BPIL1	0.92 (± 0.31)	0.51 (± 0.19)	0.48 (± 0.17)
BPIL3	0.16 (± 0.13)	0.22 (± 0.13)	0.26 (± 0.11)
RYA3	0.25 (± 0.11)	0.12 (± 0.05)	0.12 (± 0.05)
RY2G5	0.06 (± 0.03)	0.10 (± 0.04)	0.04 (± 0.02)
SPLUNC3	0.90 (± 0.35)	0.66 (± 0.23)	0.54 (± 0.18)
PLUNC	0.54 (± 0.19)	0.31 (± 0.11)	0.22 (± 0.08)
VEMSGP	0.39 (± 0.19)	0.37 (± 0.15)	0.28 (± 0.11)
LPLUNC5		0.22 (± 0.08)	

**Table 5:** dN/dS ratios (± SE) for the BSP30 and PSP genes

	BSP30B	BSP30C	BSP30D	hPSP	mPSP
BSP30A	0.57 (± 0.32)	1.90 (± 0.60)	1.13 (± 0.38)	1.13 (± 0.34)	1.33 (± 0.40)
BSP30B		1.95 (± 0.62)	1.37 (± 0.48)	1.12 (± 0.34)	1.25 (± 0.39)
BSP30C			2.73 (± 0.90)	1.33 (± 0.38)	1.54 (± 0.47)
BSP30D				1.16 (± 0.34)	1.40 (± 0.42)
hPSP					0.96 (± 0.29)

**Table 6:** Amino acid sites under positive selection

Model	Number of Parameters	Parameter Estimates ^a^	ω	Sites under positive selection
M1a	2	*þ*_0 _= 0.114 ω_0 _= 0.185	0.9067	
M2a	4	*þ*_0 _= 0.031 *þ*_1 _= 0.771 ω_0 _= 0 ω_2 _= 3.881	1.541	80, 118, 182, 198, 214 & 233
M7	2	*þ *= 0.166 *q *= 0.012	0.916	
M8	4	*þ*_0 _= 0.793 *þ *= 0.171 *q *= 0.011 ω_s _= 3.612	1.478	80, 118, 182, 198, 214 & 233

^a ^[44, 49, 50]
