# Supplementary material for: Expansion of the Bactericidal/Permeability Increasing-like (BPI-like) protein locus in cattle
Source: BMC Genomics. 2007 Mar 15;8:75. doi: 10.1186/1471-2164-8-75 (PMC1839098; doi:10.1186/1471-2164-8-75)
Supplement: Additional file 1 — Final nucleotide and amino acid sequences for the BPI-Like genes, not already in the public sequence databases. The N-terminal domain of the two-domain (all but BSP30C, BSP30D and BASE) and the full length sequences of the single-domain sequences (BSP30C, BSP30D and BASE) were used in the Phylogenetic analyses [file 1471-2164-8-75-S1.doc]

Additional file 1: Final nucleotide and amino acid sequences for the BPI-Like genes, not already in the public sequence databases, used in the Phylogenetic analyses

| Gene | Nucleotide sequence | Amino acid sequence |
| --- | --- | --- |
| BT_BPIL1 | ATGCCTGGGGCGTGTGGGCTGGGCCTGCTGCTGGCACTGATGCTGCTGCCCGTGGTCAGTGCCTCCAGGC  CGGGCACCGTGTTCAGACTCAACAAGGAAGTGCTGAGCTATGCGGCTGAAGCCGGGAAAGCCCCGCTCCA  GAATGCCCTGCAGGTCACAGTGCCGCTTTTCCTGGACGAGAGTGGAGGGGTCTTCCAGCCCACCAGGATT  CAGATTCGGAATGTCGATGTGTCCCACCTCCAGCTGACATTTGTTGCCGATTTCGGAATACGCTTGTTGG  CAGCCACCAATTTTACTTTCAAGATCTTCCGTGACCCGGAGCCCCTGCACCTGCTGCTCCCCATAGCACT  GCTGGCTGACACCTCCGTGGAGCAGGGCTCCATCGGGACCCCCGTGGTCAGCATCTCCAGCTGCTTTTCC  ATCTTCGACAAAGCCATCGTGTTTGATGGCCGCAACAGCACAGCCCCCGCGATGCTGGCCCCGCTGCAGA  GTCACATCACAGCTGTGCTGAAGAGCAAGCTGTGCCTGAGAATCTCCAACCTGGTGCAAGGCCTCAATGT  CCACCTGGGCACTTTAATTGGCCTCAGCCCTGTGGGTCCAGAATCCCAGGTCCGCTACTCCATGATTGAC  ACCCCTACCATCACCAACGACTACATTTCCTTGGATATCAACGCTGTTCTCTTCCTGCTGGGCAGACCCA  TCGTTCTGCCTGTGGATACTACCCCCTTCGTGCTGCCACAGCACATGGGCACCAAGGGTGCCATGGCAAC  CGTGGGCCTCTCCCAGGACCTGTTTGACTCTGTCATCATGCTGCTGCAGAAGGCTGGTGCGCTCAACCTG  GACGTCACAGCGCAGCTGAATTCGAGTAACAACCCGCTGAACACCTCTGTGCTGGGCCAGTTCATCCCCG  AGGTGGCCCGTCAATTCCCCGAGCCCATGCCCCTGCTGCTCAAGGTGAGGCTGGGTGCCACGCCCACAGT  CACGTTCCGCACCAACAACGCCACGCTGCAGCTGCAGCCCTTTGTGGAAGTCCTGGCCCCGACCTCCAAC  TCGGCTTTCCAGTACCTCTTCTCCCTCAATGTGGCAGTGAACCTGAGCCTCCAGCTTTCTGTGTCCAAGG  TGAGGCTTCGGGGGACCACATCTGTGCTGGGGAATGTCCAGCTCACCGTTGCCTCCTCCAATCTGGGCTT  CATTGATACCAACCATGTCCAGACACTCATGGGCGAAGTGTTTGAGAAGCCCTTGCTGGACCACCTCAAT  GCTCTCTTGGGCATGGGGATTGCCCTCCCCCACGTGGTCAATCTCCAGTACGTCAACCCTGAGATTTTTA  TCCAGGAGGGCTATGTCGTGGTGTCCAGTGGACTCTTGTACCAGCGCTGA | MPGACGLGLLLALMLLPVVSASRPGTVFRLNKEVLSYAAEAGKAPLQNALQVTVPLFLDESGGVFQPTRI  QIRNVDVSHLQLTFVADFGIRLLAATNFTFKIFRDPEPLHLLLPIALLADTSVEQGSIGTPVVSISSCFS  IFDKAIVFDGRNSTAPAMLAPLQSHITAVLKSKLCLRISNLVQGLNVHLGTLIGLSPVGPESQVRYSMID  TPTITNDYISLDINAVLFLLGRPIVLPVDTTPFVLPQHMGTKGAMATVGLSQDLFDSVIMLLQKAGALNL  DVTAQLNSSNNPLNTSVLGQFIPEVARQFPEPMPLLLKVRLGATPTVTFRTNNATLQLQPFVEVLAPTSN  SAFQYLFSLNVAVNLSLQLSVSKVRLRGTTSVLGNVQLTVASSNLGFIDTNHVQTLMGEVFEKPLLDHLN  ALLGMGIALPHVVNLQYVNPEIFIQEGYVVVSSGLLYQR |
| BT_BPIL3 | ATGCCCCTCAAGGGTCTTGCCAGGAGCAGAGCAGAGGCTGGGCACCCTGAGGCCCCAGAGGTCCAGAATG  CCATGGATGAGAGTCATATCCTGGAGAAGATGGCAGCTGAGGCAGGCCAAAATCGCCCAGGGATGAAGCC  TATCAAGGGCATCACTGAGTCAAACTCCAGCAGTAGCTTGCTGAATGATAGGTGCAAACTAGTTTCGTTG  CTTGCATCATNCAGGGTGGGCATCTTCCAGTGTGTGTCCACGGGCATGACCATCACCGGCAAGAGCTTCA  TGGGTGGGAACATGGAGATCATCGTGGTCCTGAACATCACAGCCACCAACCGGCTTCTGCAGGACGAGGA  GACGGGCCTCCCCATGTTCAAGAGCGAGGGCTGTGAGATCATCCTGGTCAGCGTGAAGACAAACCTGCCT  AGCAACATGCTGCCCAAAATGGTCAACAAGTTCCTGGACAGCACCCTACACAAAGTTCTTCCTGGTCTGA  TGTGCCCTGCCATTGATGCAGTCCTGGTGTATGTGAACAAGAAGTGGGCCAGCCTGAATGCCCCCATGCC  TGTGGGCCAGATGGGCACTGTCCAATATGTCCTGACGTCTGTACCGACCACAACACCCAGCTACATTCAA  GTGGACTTCAGTCCTGTGGTGCAGCAGCAAAAGGGCAACACCATCCAGCTTGCGGATGCCGGAGGGGCTG  AGTTCCCTGAGGACTATGCTGAAGGCTCCTCACAGCTGCTGCTCTCGGCCACCTTCCTCACAGCAGAGCT  TGCTCTTCTGCAGAAGTCCTTTAATGTAAAAGTCAAGGATACGATGATCGGTGAGCTGCCACCCCAAACC  ACTGTGACACTGGCTGGCTTCATCCCTAAAGTGGCTGAAGCCTATCCCAAGCCGAAACCCCTGGTGACCC  AGATCAGGATAAACAAGCCCCCCAAGGTCACCATGAAGACAGGCAAGAGCCTGCTGCACCTCCACGGCAC  CCTGGAGATGTTTGCTGCACGGTGGCGGGGCAAGCCGCCTGTATCCCTCTTTGTCCTGGAAACTCACTTC  AACCTGAAAATCCAGTACTCAGTTCATGAGGACCGGCTGCAGATGACCACCTCTCTGGACAGATTACTGA  GCATGTCCCGGAAGTCCTCATCAATTGGCCCCTTCAAGGAGAAGAAATTGACTGGCTTCATCACTGATTT  TCTCCAAGAAGCCTACATCCCAGTCATCAATGATGTACTCCAAGTCGGGTTCCCACTTCCTGACCTTCTG  GACATGAACTACAACCTGGCAGAGCTGGACATAACTGAGAATGCCCTGGTGCTGAACTTGAAGCTGGACT  GA | MPLKGLARSRAEAGHPEAPEVQNAMDESHILEKMAAEAGQNRPGMKPIKGITESNSSSSLLNDRCKLVSL  LASXRVGIFQCVSTGMTITGKSFMGGNMEIIVVLNITATNRLLQDEETGLPMFKSEGCEIILVSVKTNLP  SNMLPKMVNKFLDSTLHKVLPGLMCPAIDAVLVYVNKKWASLNAPMPVGQMGTVQYVLTSVPTTTPSYIQ  VDFSPVVQQQKGNTIQLADAGGAEFPEDYAEGSSQLLLSATFLTAELALLQKSFNVKVKDTMIGELPPQT  TVTLAGFIPKVAEAYPKPKPLVTQIRINKPPKVTMKTGKSLLHLHGTLEMFAARWRGKPPVSLFVLETHF  NLKIQYSVHEDRLQMTTSLDRLLSMSRKSSSIGPFKEKKLTGFITDFLQEAYIPVINDVLQVGFPLPDLL  DMNYNLAELDITENALVLNLKLD |
| BT_RYA3 | GTGACACTGACAATGTTGGGCATGTGGTCCCCGCTTCTCCTCTGGGGTCTGGTGACTCCGTGCCAGGGGC  TGCTTGAGACGGTGGGCACCCTCGCTAGGATCGACAAGGACGAACTCGGCAAAGCCATCCAGAACTCACT  GGTCGGTGGGCCCATTCTACAGAACGTGCTGGGAACAGTCACGTCTGTGAACCAGGGCCTCCTGGGCTCC  GGGGGCCTGCTCGGAGGAGGTGGCTTGCTGAGCTATGGAGGGGTTTTTGGCGTTGTTGAGGAGCTTTCTG  GGCTGAAGGTGGAGGAAGTCACACTGCCAAAGGTGTCTGTGAAGCTGCTGCCAGGGTTTGGGGTGCAGCT  GAACCTGCACACCAAGGTGGGCCTGCACGGCTCTGGCCCCCTCGGGAGCGTCCTGCAGCTGGCTGCTGAG  GTGAACGTGTCGTCGCGGGTGGCGCTGGGCGTGAGCGCGCGGGGTACGCCCATCCTTATCCTCAAGCGCT  GCAGCACGCTCCTGGGACACATCAGCCTGCTCACGGGGTTGCTACCTGCACCACTCTTTGGGGTTGTGGA  ACAGACACTCTTCAAGGTGCTGCCAGAATTGCTGTGCCCCGTGGTGGACAGTGTGCTGGGTGTGGTGAAT  GAGCTCCTGGGGGCCGTGCTGGGCCTGGTGCCTCTCGGGGCTCTTGGGTCCGTGGAATTCACTCTGGCCA  CACTGCCTCTCATCTCCAACCAGTACATAGAGCTGGATGTCAATCCCATCGTGAAGAGCGTAGCTGGTGA  CGTCATTGACTTCCCCAAGCCCCGCAACCCCGTCAAGGTGCCGCCCAAGGAGGACCACACATCCCAGGTG  ACCGTGCCTCTGTTCCTCTTTAACACCGTGTTTGGGCTCCTTCAGACCAGCGGTGCCCTTGACCTGGACA  TCACCTCCGAGCTGGTTCCCAGCAATGTCCCACTGACAACTACAGATCTGGCAGCTTTGGTCCCTGAGGC  CCTGCGGAAGCTGCCTCCAGGCCAGCAGCTCCTGCTCTCCTTGCGGGTGAAGGAAGCGCCCACAGTCACG  CTTCAGAACCACAAGGCCACAGTCTCCATCTCAGCTACCATCCATGTGCTGTCCTACTTCCCTCAGGGGG  CCCATGAAGCCTTGTTCCAGCTGAATGGGGTGATGACTTTAAATGCCCAGCTGGCTCCCTCGGCTACCAA  GCTGCACATCTCGCTATCCCTGGAACGGCTCAGCGTCCAGCTGGTGTCCTCCTCTGCTCACACCTTCGAT  GCGTCCCGTTTAGAAGAATGGCTCAGCAGTGTGGTCCGGCTAGCTTACGTGCCAAAGCTCAATGGGTTTA  GTAGGATGTTGGGGATCCCCCTGCCTAAGGTTCTCAATGTCAATTTTGCCAATGCGGCCCTGGCAATCAT  AGAGAATGCCGTGGTGCTGACCGTGCCATCCTGA | VTLTMLGMWSPLLLWGLVTPCQGLLETVGTLARIDKDELGKAIQNSLVGGPILQNVLGTVTSVNQGLLGS  GGLLGGGGLLSYGGVFGVVEELSGLKVEEVTLPKVSVKLLPGFGVQLNLHTKVGLHGSGPLGSVLQLAAE  VNVSSRVALGVSARGTPILILKRCSTLLGHISLLTGLLPAPLFGVVEQTLFKVLPELLCPVVDSVLGVVN  ELLGAVLGLVPLGALGSVEFTLATLPLISNQYIELDVNPIVKSVAGDVIDFPKPRNPVKVPPKEDHTSQV  TVPLFLFNTVFGLLQTSGALDLDITSELVPSNVPLTTTDLAALVPEALRKLPPGQQLLLSLRVKEAPTVT  LQNHKATVSISATIHVLSYFPQGAHEALFQLNGVMTLNAQLAPSATKLHISLSLERLSVQLVSSSAHTFD  ASRLEEWLSSVVRLAYVPKLNGFSRMLGIPLPKVLNVNFANAALAIIENAVVLTVPS |
| BT_RY2G5 | ATGTGGACGGCATGGTGTGTGGCTGCTCTGTCAGTGGCGGCTGTGTGTGGCGTCCGCCAAGATACAAACA  CCGTCCTCAGGGTTACCAAACACGTGCTGAGCAACGCCATCTCGGGCACCTTGCAGCAAAGTGATGCTTT  TCGCTCAGCCCTGAGAGAGGTGCCCATGGGTAAAGCTGGTGGTGATGGTGGCGGGCCCTCTCCTGGGGGC  GTCAGGGACCTCCGAACCAGCAACTACCGCAAAGCTGAGAATGCACACGGCAGCCACAGGGACCACGGGC  GGTACAGGTCTGCTGAAGGCGCGGCGCCCGTGGGCAGACTTCACCGGCGAGAGTTGAGGCCTGGAGAGAT  CCCGCCTGGTGTAGCCACTGGGGCACTGGGCCCAGGCGGTTTGCTGGGCACGGGGGGCATGCTGGCAGCT  GACGGCATCCTAGCAGGCCAAGGTGGCCTGCTCGGCGGAGGTGGTCTCCTTGGAGACGGAGGACTTCTTG  GAGGAGGGGGCGTCCTGGGCGTACTTGGCGAGGGCGGCATCATCAGCACCGTGCAGGGCATCACCGGGTT  ACGCATTGTGGAGTTGACTCTCCCTCGGGTGTCTGTGCGGCTCCTGCCTGGCGTGGGCGTCTACCTGAGC  TTGTACACCCGTGTGGCCATCAACGGGAAGAGTCTTATTGGGTTCCTGGACATCGCAGTGGAGGTGAACA  TCACGGCCAAAGTCCGGCTGACCATGGACCGCACGGGTTACCCAAGGCTGGTCGTTGAGCGATGTGATAC  CCTCCTGGGAGGTATCAAAGTCAGGCTGCTGCGAGGGCTACTCCCCAACCTTGTGGACAACTTAGTAAAC  CGAGTCCTGGCCAATGTCCTCCCTGACTTGCTCTGCCCCATCGTGGACGTGGTGCTGGGCCTTGTCAACG  ACCAACTGGGTCTCGTGGACTCTCTGATTCCTCTGGGGATACTGGGAAGTGTCCAGTACACCTTCTCCAG  CCTCCCGCTCGTGACTGGGGAATTCCTGGAGCTGGACCTCAACACTTTGGTTGGAGAAGCTGGAGGAGAC  CTCATCGACTACCCACTGGGGCGGCCAGCTTTGTCTCCCAGGCAGAAAATGCCAGAATTGCCCCCCATGG  GTGACAACACCAACTCCCAACTGGCCATTTCTGCCAACTTTCTGGGCTCGGTTCTGACTCTCCTGCAGAA  GCAAGGGGCCCTAGATATTGACATCACCGATGGCATGGTGATCCCCCCTCCACCGTGGATGGCCCTGCTC  ACCCCACCTGGTGTCTGTCCACAGGTGTTCCAGCAGTACCCCGAGTCCCGCCCCCTCACCATCAGGATCC  AGGTGCCAAACCCACCTTTGGTGACGCTGCAGAAGGACCAGGCGCTGGTGAAGGTGTTTGCCACCTCTGA  GGTCATGGTCTCCCAGCCCAATGATGTCGAGACCACCATCTGCCTCATCGATGTGGACACAGAACTCTTG  GCCATGTTTTCCGTGGAGAATGATAAGCTTATGATTGATGCCAAGCTGGACAAGGAGGGCTTAGAGGTCT  CTGGACTTGGCTGCTGGCAGGTTGGCCTCTTGGAGGTGCTGGTCGGGAAGATTTTTGACCTGGCATTCAT  GCCTGCAATGAATGCTGTGCTGGGCTCCGGTGTCCCTCTTCCCAAAATCCTCAACATCGACTTCAGCAAT  GCAGACATTGACATCTTGGAGGCAGCTCTACTGCTTGGGGAG | MWTAWCVAALSVAAVCGVRQDTNTVLRVTKHVLSNAISGTLQQSDAFRSALREVPMGKAGGDGGGPSPGG  VRDLRTSNYRKAENAHGSHRDHGRYRSAEGAAPVGRLHRRELRPGEIPPGVATGALGPGGLLGTGGMLAA  DGILAGQGGLLGGGGLLGDGGLLGGGGVLGVLGEGGIISTVQGITGLRIVELTLPRVSVRLLPGVGVYLS  LYTRVAINGKSLIGFLDIAVEVNITAKVRLTMDRTGYPRLVVERCDTLLGGIKVRLLRGLLPNLVDNLVN  RVLANVLPDLLCPIVDVVLGLVNDQLGLVDSLIPLGILGSVQYTFSSLPLVTGEFLELDLNTLVGEAGGD  LIDYPLGRPALSPRQKMPELPPMGDNTNSQLAISANFLGSVLTLLQKQGALDIDITDGMVIPPPPWMALL  TPPGVCPQVFQQYPESRPLTIRIQVPNPPLVTLQKDQALVKVFATSEVMVSQPNDVETTICLIDVDTELL  AMFSVENDKLMIDAKLDKEGLEVSGLGCWQVGLLEVLVGKIFDLAFMPAMNAVLGSGVPLPKILNIDFSN  ADIDILEAALLLGE |
| MM_RY2G5 | GCAACAGACCTCTGAGGATTCACTTGCACACCCCCTCAAGGGAAAGCAACATCAGTATGTGGACTGCGTG  GTGTGTGGCTGCTCTGTCAGTGGCAGCTGTGTGTGGTATCCGCCAAGACACAACCACAGTCCTCAGGGTT  ACAAAAGATGTGCTGGGCAATGCCATCTCAGGCACAATTCAGAAGAGTGACGCCTTCCGCTCCGCCCTGA  GGGAGGTGCCCGTGGGTGTCGGTGGTGTCCCGTACAACGACTTCCATGTCCGAGAGCCTCCCCCCAAATA  CACCAATGGCAGACAGCTTGGTGGCAATTACAAATATGGTCACATTAAGGCGAACGACAATAGAGCTCAG  CTGGGGGGCAAGTACCGCTATGGGGAGATCCTGGACTCCGATGGGAGCCTCAGGGACCTACGGCATGAAG  ACTACCGTCCTCCAGACAGTGCCTACCACCGCGGCTCTGGGCGGTACAGATCCGCCGCAGACTCATCCTC  GGTGGGCAGGCTCTACCGGCGCGAACTGAGGCCTGGAGAGATCCCAGCTGGTGTGGCCACTGGGGCCCTG  GGCCCCGGAGGTCTGCTGGGCACAGGGGGCATGCTGGCAAATGAAGGCATTCTGGCAGGCCAAGGGGGCT  TGCTTGGTGGAGGTGGTCTTCTTGGAGATGGAGGACTTCTTGGAGGAGGAGGTGTGCTCGGTGTGCTGGG  TGAAGGAGGCATCCTAAGCACCGTACAAGGCATCACTGGGTTGCGCATCGTGGAACTTACCCTTCCCCGG  GTGTCCGTGAGACTCCTGCCCGGTGTGGGTGTCTACCTGAGCTTGTACACCCGTGTGGCCATCAACGGAA  GGAGCCTCATCGGCTTCCTGGATATTGCAGTGGAGGTAAACATCACGGCCAAAGTTCGGCTGACCATGGA  CCGCACAGGCTACCCACGGTTGGTCATTGAGCGCTGTGACACCCTCCTGGGAGGTATCAAAGTCAAGCTG  CTTCGAGGGCTTCTCCCCAACCTGGTGGACAACTTAGTGAACCGAGTGCTGGCCAATGTACTCCCTGACC  TGCTCTGCCCCATTGTGGATGTGGTATTGGGTCTTGTCAATGACCAGCTGGGTCTCGTGGACTCTTTGGT  GCCTCTGGGAATACTTGGGAGTGTGCAATACACTTTCTCCAGCCTTCCACTGGTGACTGGGGAATTCCTT  GAGTTGGACCTTAATACTCTAGTTGGGGAGGCAGGAGGTGACCTCATTGACTACCCCCTGGGGCGGCCAG  CTATATTGCCCAGGCCACAGATGCCAGAACTACCCCCCATGGGCGACAACACCAACTCCCAGCTGGCCAT  CTCAGCCAATTTCCTGAGCTCGGTGCTGACCATGCTGCAGAAGCAAGGTGCAATGGACATTGACATCACT  GATGGCATGTTTGAAGATCTCCCTCCACTTACCACTTCCACACTGGGGGCCTTGATTCCCAAGGTGTTCC  AGCAATACCCGGAATCCCGCCCACTCACCATCAGGATCCAGGTGCCCAACCCTCCAACTGTGACACTGCA  GAAAGACAAGGCGCTGGTGAAGGTGTTCGCCACCTCTGAAGTTGTGGTCTCCCAGCCCAACGATGTAGAG  ACCACTATCTGCCTCATCGATGTGGACACAGACCTCTTGGCTTCATTTTCTGTGGAAGGAGATAAGCTCA  TGATCGACGCCAAGCTGGATAAGACCAGCCTCAACCTCAGAACCTCAAACGTGGGCAACTTTGATGTGTT  CATCTTGGAAATGTTGGTTGAGAAGATCTTTGACCTAGCGTTTATGCCTGCAATGAATGCTATACTGGGT  TCTGGAGTCCCCCTGCCCAAAATCCTCAACATTGACTTCAGCAACGCAGACATTGATGTGTTGGAGGACC  TTCTGGTGCTGAGCACATGAGTGACAAAGGTAGATGACATGAGAAGAAGTCAACCATGTTCAGAGAGGGT  GGTCCTAGACACCGGCCCTGGTCCCAAGTCTGTTCAGCCTGTATCACGGGGCCTGCGCCAACCCTCCCAC  CCCTTCCCCTTCCTGCCCTTAACCTGGGAAGGATCCAGCCACTCCCCATTGTCCAACAATGCCATGGTCA  GGACAGCCTGGGGGCTGAGGTCTTGTGCTTTCAATAAAACATTTCACTTTCCCTGCAAAGAAAG | MWTAWCVAALSVAAVCGIRQDTTTVLRVTKDVLGNAISGTIQKSDAFRSALREVPVGVGGVPYNDFHVRE  PPPKYTNGRQLGGNYKYGHIKANDNRAQLGGKYRYGEILDSDGSLRDLRHEDYRPPDSAYHRGSGRYRSA  ADSSSVGRLYRRELRPGEIPAGVATGALGPGGLLGTGGMLANEGILAGQGGLLGGGGLLGDGGLLGGGGV  LGVLGEGGILSTVQGITGLRIVELTLPRVSVRLLPGVGVYLSLYTRVAINGRSLIGFLDIAVEVNITAKV  RLTMDRTGYPRLVIERCDTLLGGIKVKLLRGLLPNLVDNLVNRVLANVLPDLLCPIVDVVLGLVNDQLGL  VDSLVPLGILGSVQYTFSSLPLVTGEFLELDLNTLVGEAGGDLIDYPLGRPAILPRPQMPELPPMGDNTN  SQLAISANFLSSVLTMLQKQGAMDIDITDGMFEDLPPLTTSTLGALIPKVFQQYPESRPLTIRIQVPNPP  TVTLQKDKALVKVFATSEVVVSQPNDVETTICLIDVDTDLLASFSVEGDKLMIDAKLDKTSLNLRTSNVG  NFDVFILEMLVEKIFDLAFMPAMNAILGSGVPLPKILNIDFSNADIDVLEDLLVLST |
| BSP30C | GCAGTGGTTGTGTATCTTTGTGCAAGCTGCTCTAGCTTCCTGACCTGGGCTGCCCACCTGTCAGAGAGCA  GATAGCCACGTCTACCTTCAAGGACTGCTGAGTGTAAGACTGATAGGTGCTCATTAAAGATCCCACTTCC  CTGTGGGTGACTCTCACTCCTGTTCCTCCCGTGACTGTCCAGGACAATATGCTTCCACTTTGGAGACTTG  TTCTCTTGTGTGGCCTGCTCACTGGGACGTCAGCGTCTCTTCTTGACAACAACGTTGTGAGAGAGCTGCA  ATCTGCTCTTAGAAAGGAGCTCGAGACGGATGACAGTGCATCTAAACCTGTCCTTGAGAAAGTGAAGGCT  GATTTTGAGTTGCTCCAGGATTTCACATGTTTGGAGATGATGGCTGTGAAGGAGGTACTTCCCGAGAAGA  TCCAGGATGCTGAGATCGTGTTGGACAAGGACAAGAGTAACAATCGTCAGCTACTTGTCAGGTGTTTACG  GTTGACAATCAGGAGCATCAGCATCGGGAATATCACATTCCAAGTGACTCCTGGAGGCACAAGCATTAAC  CTGAGTATCTCCATCACTGCTAAGGTCACCCTGACCCTGCCTCTGTTGGGTGCGGTCATTGACTTGACCC  TCAACTTTGTCCTACAGAGAAGTATCAGCTTTAAAATTGATGAAGCTGGTACCTTAATGGTGGTCCTGGG  AGAATGCACCTACACACCAGCCAAGATATCACTCCCCTTCGTGAACAGTTCTGTCTCCAGCCTCACTGGA  CTGATGAGCAATATCAGAAAGACTGTGACCACACTCGTGAATCTGGTGGAGACCTACATAGTGAAGTATG  TGCTGTGCCCACGAATTGGCACCATCATCAGCTCCTTGAATGAGAATTTGGTTAATAACCTCAACGATAT  ACTTCAGAAAACCGCACAACAAATTGTAAACTGAAGAGAACAGATGAGAAAAACCACAGTGATGCCCGCC  TGCTGATTGGTCCCCGGGGACTTGACTGAATCTCTGTACCATCTCCCTCTGGGACGGTCGGTGCTGCCAC  CATCCCCCAGGAGTGACAACTGAGCCCAGTCAAGGACACTCTCAGATACGCCTCCTCACAGTCAGGATAC  CCTATGCTACTCGCCCTCCACCCCCAGCAATAAAAAGGCATTTCTGCACCTTCAAAAAAA | MLPLWRLVLLCGLLTGTSASLLDNNVVRELQSALRKELETDDSASKPVLEKVKADFELLQDFTCLEMMAV  KEVLPEKIQDAEIVLDKDKSNNRQLLVRCLRLTIRSISIGNITFQVTPGGTSINLSISITAKVTLTLPLL  GAVIDLTLNFVLQRSISFKIDEAGTLMVVLGECTYTPAKISLPFVNSSVSSLTGLMSNIRKTVTTLVNLV  ETYIVKYVLCPRIGTIISSLNENLVNNLNDILQKTAQQIVN |
| BSP30D | GTGTCCGGACAAGATGTTTCAGCTTTGGAAACGTGTTCTCTTGTGCGGCCTGCTCCCTGGGACCTCAGCG  TCTCTTCTTGACAGTCTTGGCAAGGATGGTCTGAGGAAGCTGAAATCTGGTCTTGAGAAAGGACTTGACA  ACCTTGACACTATCTTGGAGCAATTGAAGACTGCTGAGGCGGACCCAGAGAAGACTGAAGAAGCTAAGAG  CCTGTTGGAACAACTCATTTCTGGAATTTTTGAAGTAGTGTACAGGCTTACGGGGGTGAATATCAGTAAC  CTGCACATCCTGAATATCACATTAGAACCAGCTTCTGATGGCAAAGGTGCTACCTTGAAAATCCCCATCA  CTGCTGAAGTCAAAGTGAACCTGCCTGTATTGGGTGAGATTGTCGACTTGGCCCTCAACTTGGTCCTCCA  GTATAGTGTCAGCGTTGAAACTGATGAAGAGACTAAAGTCTCCAAGGTGGTCGTGGAAGAATGCAGGAGC  GATCAACAGAGCACCAAACTCACCATGCTGGGCAGGCGCATTGGACTGCTCACTGAGGTTGTGGACTTTG  CAATCAACCTCGTGAATGAAGTATTGTCCCTGGTAACGCATTACGAGCTGTGCCCACTGGTCCGCAGTTT  TTTTGAAAGCCTGGATGCAGATTATCTTAAGAACCACATCACTAAGTCACAGCTTGGGGAAATG | CPDKMFQLWKRVLLCGLLPGTSASLLDSLGKDGLRKLKSGLEKGLDNLDTILEQLKTAEADPEKTEEAKS  LLEQLISGIFEVVYRLTGVNISNLHILNITLEPASDGKGATLKIPITAEVKVNLPVLGEIVDLALNLVLQ  YSVSVETDEETKVSKVVVEECRSDQQSTKLTMLGRRIGLLTEVVDFAINLVNEVLSLVTHYELCPLVRSF  FESLDADYLKNHITKSQLGEM |
| HS_SPLUNC3 | CCCAGGGTTCCACATGTTGCTCTCCCAATGTGAGCAAGCCCTGGTGGCAGCGCCAGGGTCCAGTGCAGCC  CCTCCCCACAGCATGCTGGGGGCTAATTCTGATGTCATCTTTCTGCAGAAAACCATTAGACCATCCCTCC  AGACTGCCACCCTCAAAGCCGTCTGCCCAGGCCCCATCTGACACTCTTGACATCTGCAGGTCCCAGACCC  TATGATGTGTCCACTCTGGAGGCTCCTCATCTTCCTCGGGTTGCTGGCCTTGCCCTTGGCACCACACAAG  CAGCCTTGGCCTGGCCTGGCCCAAGCCCACAGAGACAACAAATCCACCCTGGCAAGAATTATTGCTCAGG  GCCTCATAAAGCACAACGCAGAAAGCCGAATTCAGAACATCCACTTTGGGGACAGACTGAATGCCTCAGC  ACAAGTGGCCCCAGGGCTGGTGGGCTGGCTAATCAGCGGCAGGAAACACCAGCAGCAGCAAGAGAGCAGC  ATCAACATCACCAACATTCAGCTGGACTGTGGTGGGATCCAGATATCATTCCATAAGGAGTGGTTCTCGG  CAAATATCTCACTTGAATTTGACCTTGAATTGAGACCGTCCTTCGATAACAACATCGTAAAGATGTGTGC  ACATATGAGCATCGTTGTGGAGTTCTGGCTGGAGAAAGACGAGTTTGGCCGGAGGGATCTGGTGATAGGC  AAATGCGATGCAGAGCCCAGCAGTGTCCATGTGGCCATCCTCACTGAGGCTATCCCACCAAAGATGAATC  AGTTTCTCTACAACCTCAAAGAGAATCTGCAAAAAGTTCTCCCACACATGGTAGAAAGTCAGGTATGTCC  TCTGATCGGTGAAATCCTCGGGCAGCTGGATGTGAAACTGTTGAAAAGCCTCATAGAACAGGAGGCTGCT  CATGAACCAACCCACCATGAAACCAGCCAACCCTCTGCATGCCAGGCTGGAGAGTCCCCCAGCTGACTTC  TGCTGATCAGAAGGAAAGTCCACATCTTGCAACCTTAAGTCTCCCTTAGAGTGGGGCTTCTGCTACCCTA  AAAACTTTACCCCAGGCTCTGTGGACATACCATCCTCTCCTACAATAAACTCTAGCTCTGAAGGGTGCAC  AGGTCCCTCCCACCTGGGCCCTGGGGTTTAGGTCTAGAAGTCAG | MMCPLWRLLIFLGLLALPLAPHKQPWPGLAQAHRDNKSTLARIIAQGLIKHNAESRIQNIHFGDRLNASA  QVAPGLVGWLISGRKHQQQQESSINITNIQLDCGGIQISFHKEWFSANISLEFDLELRPSFDNNIVKMCA  HMSIVVEFWLEKDEFGRRDLVIGKCDAEPSSVHVAILTEAIPPKMNQFLYNLKENLQKVLPHMVESQVCP  LIGEILGQLDVKLLKSLIEQEAAHEPTHHETSQPSACQAGESPS |
| BT_BASE | ATGCTGAAGGTCTCCAGCCTCTTCATTCTCCTCTGTGGACTGCTTGCTTTATCATCTGCGCAAGAAGTCC  TGTCTGAAGTTTCTTCCCAGATCAACGATGTCTTGACTAAAGAACTCCTCAGCGTGGGCTTTCTCCCGAG  CCTGCAGAATATTGACCTTCAAGAGTCATTGCAGAATGTCTTCAGCCAACCAGCTGGCCTGCTGGACATC  AACAGTGACAGCAATGTTGTGGTAGAACTAGAAGACCCGCGCCTCATTCAGGTCTTCCTCCAGGACTCTG  TCAACAACAAGGAAGCCGGATTACTGGTAGCACTGGCATTTTCTGTACATACAAAGCTTCCAGTTCTTAA  TCCCCTCATATTTCAAGTGAGGACAAATATGAAAGTCCAGCTGCGTTTGGAGAAGGATGTAAATGGCATA  TACCGGCTCACCTTCGGGCATTGCAGGCTCATTCCTGAGAGTGTTCGGATCCAATCTGGAAGTCTAACCA  GTCGGATACCAAATTTTGTTTGGGAATATGTGGAGATAACTATAAAAAACCTGATCATCAATAATTTGGG  AGAAAAAGTATGCCCCTTCATCAATTCATGGCTCTATAACCTGAACCCACAGGTGGCTAATGAGCTGATG  AACTGGCTGCTGCAGCAGAGTGGGTACCAAGCCGCCATTGAAATAGCTTCAAAGTGA | MLKVSSLFILLCGLLALSSAQEVLSEVSSQINDVLTKELLSVGFLPSLQNIDLQESLQNVFSQPAGLLDI  NSDSNVVVELEDPRLIQVFLQDSVNNKEAGLLVALAFSVHTKLPVLNPLIFQVRTNMKVQLRLEKDVNGI  YRLTFGHCRLIPESVRIQSGSLTSRIPNFVWEYVEITIKNLIINNLGEKVCPFINSWLYNLNPQVANELM  NWLLQQSGYQAAIEIASK |
| BT_LPLUNC5 | ATGCTGCTGTTCTGGGGGTCACTGCTCTGCTGGGGGCTGCTGCCCCAGGTCCAAGGTGAAGCCCAGCACT  TTGTCTTCATGCGCATCAGCAAGGATCAGCTGGAGACAGACATTTCAGACCTGCTCTTTAAACACCGCGT  CCTGGACCGGGTGACCAGGATCCCTGTGAGGGGGGACGCAGGTGAAGGCATTGCCATCTTGGACCACATG  CCCTTCGTAAGAAAAGGTCTCTCCAAGAAGAGCACTGGGCTTGACCTGCCACTGATCAGGGGTCTGCTCT  TGGGACAGAGCCTCTCCCCGCTGGGGGAGCTGCTCCAGCTGGGCGGGTTGGTCATAGAAGATTCTGAAGG  ACCCGAGGTCACCTTACAAGTCCTGAGTGACAGCCTGCTGCAGGTCACATTGCGAAGCAAACTGTACCTC  TCACTCCAGAGGATCCTGCGGCTCAGAGTCATCAAGAACATCCGCATCGGGGCTCGGCTGGAGCAGACGG  GGAACAAGACCCACGTGGCCTTTGAGGAGTGCCACACCCCACCAGGGTACCTGAGCATCGAGGTTCTGGA  GCAGATGAACCCCCTCCTGGTGAACGAAGCTCTGCGGTTGGTGACAGATGTCCTGGACGAGGCATTGCCC  TTCCTCCTGCAGAAAATAGTGTGCCCCACTGCCACAACCCTGCTCAACTCGCTGCTGGAAGACCTGTTAC  ACATCACTCTGCCCCCAGCCAGCTCAGGCCCAGAAGACTTCCAGTACTATGTCACCACCACAGAATTCAC  GGAAAAGGCCATCCTGATGAAAGTCCAGCTTGTGACCCCCTGCAGTCCAGGCCAGAGAGTCCCAAGGCCT  GGCCACCTGGCCCCCAAGCCCCTCCCCAAATTAGCCCAGGGCAGCCTGGCAGACCTGGTCTTTTCACTGG  AAACCTACAACGACATCCTGTCCTGCCTGTACACCAGCAAGGAGATCCATGTGAACCCCCAGGACCCCGT  GGCTGCCAACCTCAACCAGCTATTGTCACTGAGCGTACTGGAGCCTGAGCCCAAGACTTCTGATCAGTCT  AGAGGGAACGTGGGACTGACCATCAGCATCCCTGACCCTCCCATCGTCCACCTTGATGGCCACACAGCCT  CCGTCATCCAGCCAGGCTCCCTGGTGCTGGCAGGGTCCAGCAACACCTCCCCCGTCTCAGTTTCCTGGAA  ACTCCTTTCAAATGCTGTGTTTTCCTCGAAAAATCGGGAATTAAAACTCCAGTTCACTCCAAACAGCATT  ACAGTCATCCTGGGCCCTTACCCCGCTGGCCTTGAAAAGCAGAAAGAATGTCTGGAGGCCTTTCTCTTCG  AGCTTCTGAACTGGAGGGTCCTGCCCCATCACAACAACCTGCTCAGAGAACAAGGCATCGACCAGGCACG  AATGGAGCCCTCCGAGGTAAATGCTGGGAAAAGGAGCTTCCCGGGGCCCACGTGGGAGGTCAGAGCTATA  GGAGCCATGGGGTCCTACAGCCCCTCTGTATTCAGTGTCTTCGACAGTGGAAGTGACTCAGAAAATGGAA  AGGGGGCTTGA | MLLFWGSLLCWGLLPQVQGEAQHFVFMRISKDQLETDISDLLFKHRVLDRVTRIPVRGDAGEGIAILDHM  PFVRKGLSKKSTGLDLPLIRGLLLGQSLSPLGELLQLGGLVIEDSEGPEVTLQVLSDSLLQVTLRSKLYL  SLQRILRLRVIKNIRIGARLEQTGNKTHVAFEECHTPPGYLSIEVLEQMNPLLVNEALRLVTDVLDEALP  FLLQKIVCPTATTLLNSLLEDLLHITLPPASSGPEDFQYYVTTTEFTEKAILMKVQLVTPCSPGQRVPRP  GHLAPKPLPKLAQGSLADLVFSLETYNDILSCLYTSKEIHVNPQDPVAANLNQLLSLSVLEPEPKTSDQS  RGNVGLTISIPDPPIVHLDGHTASVIQPGSLVLAGSSNTSPVSVSWKLLSNAVFSSKNRELKLQFTPNSI  TVILGPYPAGLEKQKECLEAFLFELLNWRVLPHHNNLLREQGIDQARMEPSEVNAGKRSFPGPTWEVRAI  GAMGSYSPSVFSVFDSGSDSENGKGA |
