# Supplementary material for: Expansion of the Bactericidal/Permeability Increasing-like (BPI-like) protein locus in cattle
Source: BMC Genomics. 2007 Mar 15;8:75. doi: 10.1186/1471-2164-8-75 (PMC1839098; doi:10.1186/1471-2164-8-75)
Supplement: Additional file 2 — List of public domain nucleotide sequences (The N-terminal domain of the two-domain and the full length sequences of the single-domain sequences were used for the phylogenetic analysis). [file 1471-2164-8-75-S2.doc]

Additional file 2: Human and Mouse BPI-like genes

| Gene | HS GenBank nucleotide & amino acid accession nos. | MM GenBank nucleotide & amino acid accession nos. |
| --- | --- | --- |
| BPIL1 | NM_025227, NP_079503 | NM_025631, NP_079907 |
| BPIL3 | NM_174897, NP_777557 | NM_199303, NP_955007 |
| RYA3 | NM_182658, NP_872599 | NM_194357, NP_919338 |
| RY2G5^1^ | NM_182519, NP_872325 | Genscan predicted sequence |
| PSP | NM_080574, NP_542141 | NM_008953, NP_032979 |
| BASE | NM_173859, NP_776258 | - |
| SPLUNC3^1^ | Genscan predicted sequence | NM_028528, NP_082804 |
| PLUNC | NM_130852, NP_570913 | NM_011126, NP_035256 |
| VEMSGP | NM_033197, NP_149974 | NM_153418, NP_700467 |
| SPLUNC5 | - | NM_025990, NP_080266 |
| LPLUNC5 | - | BC018465, AAH18465 |
| SPLUNC6 | - | XM_622305, XP_622305 |

^1^ See additional file 1
